# RCC2 promotes prostate cancer cell proliferation and migration through Hh/GLI1 signaling pathway and cancer stem-like cells

**DOI:** 10.1186/s13062-023-00439-w

**Published:** 2023-11-27

**Authors:** Shenghan Wang, Zhentao Lei, Wei Liu, Jie Xiong, Yuqiang Shi, Lin Yang, Qiang Gao, Kai Le, Bao Zhang

**Affiliations:** 1 Department of Urology, Aerospace Center Hospital, No.15, Yuquan Road, Haidian District, Beijing, 100049 China; 2 Department of Urology, Zhongnan Hospital of Wuhan University, Wuhan, China

**Keywords:** RCC2, Prostate cancer, GLi1, Stem activity

## Abstract

**Background:**

Regulator of chromosome condensation 2 (RCC2) was a telophase disk-binding protein on mitosis, and functions as an oncogene in many human cancers. However, its role on prostate cancer (PCa) was unknown. The goal of this study is to explore the function of RCC 2 on PCa development.

**Methods:**

The expression of RCC2 and its methylation level, its correlation with lymph node metastasis or disease-free survival (DFS) was analyzed using TCGA database. The effect of RCC2 on PCa cell proliferation, migration and invasion were detected using CCK-8, cell colony formation, Transwell and wood healing assays. RNA-seq and GSEA analysis were used to search the downstream genes and pathways of RCC2 in mediated PCa progression. Western blot was used to detect the proteins in PCa cells transfected with indicated siRNAs or plasmids.

**Results:**

RCC2 had high expression and low promoter methylation level in PCa, and its expression was correlated with regional node metastasis and disease-free survival. Cell proliferation, migration, invasion and EMT of PCa cells in vitro were greatly enhanced after RCC2 overexpression, while the RCC2 knockdown suppressed these processes. RNA-seq and GSEA results showed the Hedgehog signaling regulator Gli1 and Gli3 were involved in RCC2 knockdown DU145 cells. Gli1 was also a marker of cancer stem-like cells (CSCs). Mechanistically, RCC2 induced cell growth, EMT, CSCs markers through Gli1; inhibiting Gli1 expression using siGli1 or GLI inhibitor suppressed cell progression in vitro and tumor growth in vivo.

**Conclusion:**

In summary, RCC2 promoted PCa development through Hh/Gli1 signaling pathway via regulating EMT and CSCs.

## Introduction

Prostate cancer (PCa) is the second most common cancer in men and the fifth cause of cancer-related deaths worldwide [1]. The treatment of PCa relies on grade, stage, and age and varies from active supervision of a mixture of operation, radiation, chemotherapy, and Androgen deprivation therapy (ADT) [2]. Operation (prostatectomy) or radiotherapy can cure most PCa patients of low-risk disease with five-year progress-free survival rates above 90%. However, the survival for high-risk PCa patients remains poor [3], with a 5-year survival rate of 30% [4]. Cancer stem-like cells (CSCs) and epithelial-mesenchymal transition (EMT) are two significant and closely-linked cellular programs of PCa progression [5]. Understanding the underlying mechanisms of cancer progression is essential for drug development in PCa.

Regulating agent of chromosome condensation (RCC) 2 was first observed as a telophase disk-binding protein [6], indicating its effect on mitosis. During mitosis, the right assembly of the mitotic spindle and stimulation of core mitotic proteins require RCC2 [7]. Recently, many reports showed that RCC2 is involved in many human cancers, functioning as an oncogene to promote cancer progression, tumorigenesis, and drug resistance [8–11]. Wang et al. [9] found that RCC2 expression stimulates ER-positive breast tumorigenesis via promoting cell migration and inhibiting apoptosis. Chen et al. [12] reported that RCC2 could promote metastatic behaviors and cisplatin resistance in HCC cells.

Currently, there were no reports about the function of RCC2 in PCa. In this study, we aim to explore the possible role of RCC2 in PCa progression, in order to find new ways for prostate cancer therapy.

## Methods

### Samples from the cancer genome atlas and GEO

There were 549 samples gathered from The Cancer Genome Atlas (TCGA) database, including 497 PCa samples and 52 normal prostate tissue samples. The expression of RCC2 and its promoter methylation level were analyzed. According to the regional lymph node metastasis, 497 PCa samples divide into 345 N0 stage and 79 N1 stage. The disease-free survival (DFS) was analyzed using the Kaplan-Meier approach based on the mean value of RCC2. Moreover, we verified the expression of RCC2 expression between Normal and Tumor on GEO_46602.

### Cell culture and cell transfection

American Type Culture Collection offered human PCa cell lines, DU145 and PC3, and normal prostate cell RWPE-2. Cell cultivation was performed in Dulbecco’s modified Eagle’s Medium (DMEM), and 2 mM L-glutamine, 10% fetal bovine serum, and 1 mM sodium pyruvate were added. The cells were incubated in a humidified incubator at 37℃ with 5% CO_2_.

PCR with the specific primers *Asc* I-Fex and *Kpn* I-Rex were used to obtain the full coding area of the RCC2 gene. After verification with sequencing analysis, the insertion of incurred PCR product was made into the *Asc* I/*Kpn* I places of a pcDNA 3.1 (+)-RFP expression vector. PolyJet™ In Vitro DNA Transfection Reagent was used to transfect the RCC2-overexpressing plasmid into DU145 and PC3 cells based on the guidelines of the producer.

siRNAs targeting RCC2 were synthesized by Hippotech (Huzhou, China) and were transfected with Lipofectamine RNAiMAX. The sequences were as follows: siRCC2#1 5’- GCAAUCUCGGUCAGAAUUUTT-3’; siRCC2#2 5’-GCGAGUGGCCAUCUUCAUUTT-3’; siGli1, 5’- AAACGCUAUACAGAUCCUA-3’ siCtrl 5’-UUCUCCGAACGUGUCACGU-3’.

### Cell proliferation assay

For the CCK-8 assay, the 2-hour incubation of the transfected DU145 and PC3 cells was made with the Cell Counting Kit-8 solution. Absorbance was measured at 450 nm using a spectrophotometer.

For the cell colony formation assay, cells were cultivated under normal situations for two weeks after seeding in 6-well plates (800 cells/well) in triplicate. After washing and staining with 0.1% crystal violet, colonies were counted with an inverted microscope.

### Sphere formation assay

Indicated 2000 DU145 or PC3 cells were grown in low attachment 6-well plates and cultured in sphere formation medium (DMEM/F12 supplemented with 20ng/ml EGF, 20ng/ml bFGF, B27 supplement, N2 supplement). Numbers of spheres were counted under an optical microscope after 1 ~ 2weeks and photographs were taken.

### Cell migration and invasion assays

A 24-well transwell chamber was used to make migration and invasion assays. The counting and placement of cells were made in the upper chamber of the well, and 500 µL of medium containing 20% serum was added to the lower chamber. The cells were removed using a cotton swab after incubating for 24 h, and the remaining cells were fixed with 4% paraformaldehyde for 20 min. Optical microscopy was used to discover the migrated cells after staining with 0.1% crystal violet.

Similar steps were made for the invasion assay, except the upper chamber was coated with equal contents of Matrigel melted at 4 °C and diluted with serum-free medium. The cells were then incubated at 37 °C for 48 h before cell counting.

### Wound healing assay

After seeding in a 6 cm culture dish, DU145 and PC3 cells were cultivated to > 90% confluence. A p200 pipette tip was used to scratch the cell monolayer to paint five parallel cell-free lines at the bottom (about 1 mm in width). While removing the original medium, phosphate buffer saline (PBS) was used to wash the cells. Wound healing was imaged at 1, 2, 3, and 4 days after scratching.

### Western blot

Cells or tissues were collected and put into RIPA lysis buffer on ice. The cell lysates were centrifuged for 10 min at 13,000 rpm to obtain the supernatant. A BCA assay kit was used to measure the concentration of total protein. After separation by 10% sodium dodecyl sulfate-polyacrylamide gel electrophoresis, 20 µg of total protein was transferred onto polyvinylidene difluoride membranes. For blocking, the membranes were incubated with PBS containing 0.1% Tween-20 and 5% nonfat milk. Antibodies were added and incubated overnight at 4 °C to test for RCC2 (ab229153, Abcam), GLI1 (MBS846102, MyBioSource), GLI3 (K107300P, Solarbio), SOX2 (K009480P, Solarbio), Nanog (ab21624, abcam), CD44 (K007092P, Solarbio), Snail (ab216347, abcam) and GAPDH (ARG65680, Arigobio). The concentrations of these primary antibodies were recommended by the manufacturer. After three washes, relevant secondary antibodies conjugated with horseradish peroxidase were used to hybridize the membrane. Enhanced Chemiluminescence kits were employed to detect the immune signals. The ImageQuant 5.2 software was used for quantitative analyses.

### RNA-seq and GSEA analysis

The total RNA in RCC2 knockdown DU145 cells was extracted using Trizol reagent (Invitrogen) and subjected to library establishment for transcriptome analysis using the ABI SOLiD platform. The differential expression genes (DEGs) were obtained with a false discovery rate (FDR) estimate below 0.1 and a fold variation above 2. The signaling pathways in DEGs were enriched using Gene set enrichment analysis by applying the Pathway Interaction Database [13] and Gene Set Enrichment Analysis (GSEA) version gsea2-2.0.14 [14].

### Animal studies

Beijing Vital River Laboratory Animal Technology Co., Ltd offered male BALB/c nude mice which adapted to the experiment environment for two weeks. After being suspended in 200 µl PBS, 2 × 10^6^ DU145 cells transfected with pcDNA3.0-RCC2 over-expressed plasmid or GANT61 (GLI inhibitor) were injected subcutaneously into the mice axillae. Tumor volumes were measured with calipers every three days using the formula (length×width^2^)/2. In addition, tumor weight was measured after sacrificing the mice. Tumor tissues were collected for the protein expression of RCC2, Gli1, SOX2, Nanog, and CD44 using western blot. Based on ethical norms and national guidance, all animal experiments were approved by the Animal Ethical and Welfare Committee.

### Statistical analysis

The data was presented as “mean ± standard deviation” and analyzed using SPSS 20.0 software and GraphPad Prism 6 software. Student’s t-test or one-way analysis of variance (ANOVA) was used to analyze the statistical comparisons between group comparisons. P < 0.05 was considered statistically significant.

## Results

### RCC2 is highly expressed in PCa and indicates poor DFS

**Fig. 1:**
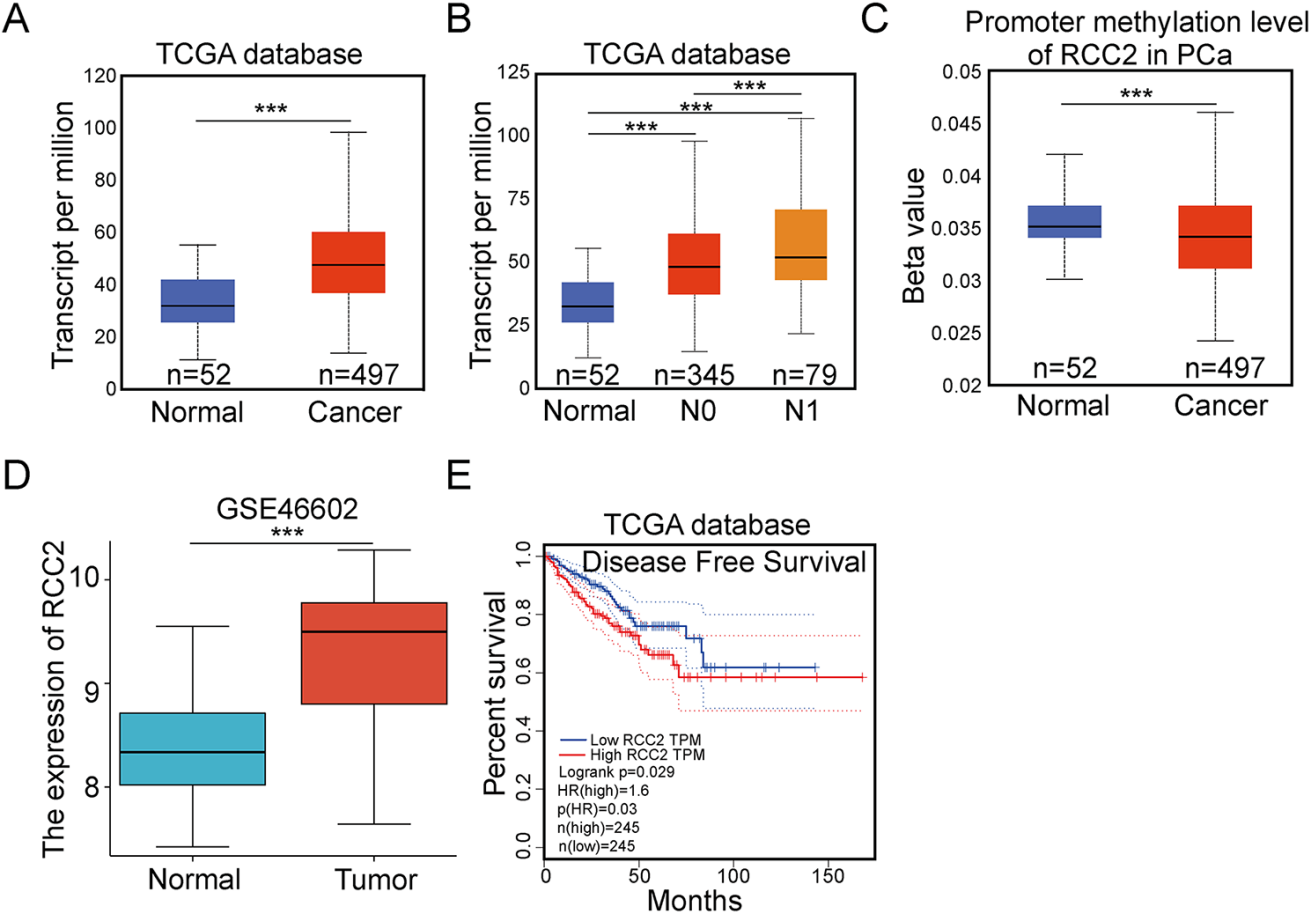
RCC2 is highly expressed in prostate cancer and indicates poor DFS. **A** RCC2 mRNA expression was compared between 497 prostate cancer samples and 52 normal prostate samples from The Cancer Genome Atlas (TCGA) database. **B** RCC2 mRNA expression was compared between PCa samples with different regional lymph node metastasis. **C** The promoter methylation level of RCC2 in PCa and normal samples from TCGA. **D** RCC2 mRNA expression was compared between prostate cancer samples and normal samples from GEO46602 database. **E** DFS between PCa patients with high RCC2 expression compared to low RCC2 expression. ***P＜0.001

To investigate the effect of RCC2 on PCa, we first determined RCC2 expression in 497 PCa tissue samples and 52 normal prostate tissue samples from The Cancer Genome Atlas Data. The findings demonstrated the up-regulation of RCC2 in PCa tissues (Fig. 1A), a significant increase in the expression of RCC2 in PCa samples with N0 and N1 (Fig. 1B), and a reduction of the promoter methylation level of RCC2 in PCa samples compared to normal ones (Fig. 1C). Moreover, we also identified that the RCC2 was high expressed on PCa samples based on the GEO 46,602 (Fig. 1D). Meanwhile, based on TCGA database, PCa patients with high RCC2 levels had poor DFS using Kaplan-Meier survival analysis (Fig. 1E). These results indicated that RCC2 may exert an effect on prostate cancer.

### RCC2 promotes cell propagation of prostate cancer

**Fig. 2:**
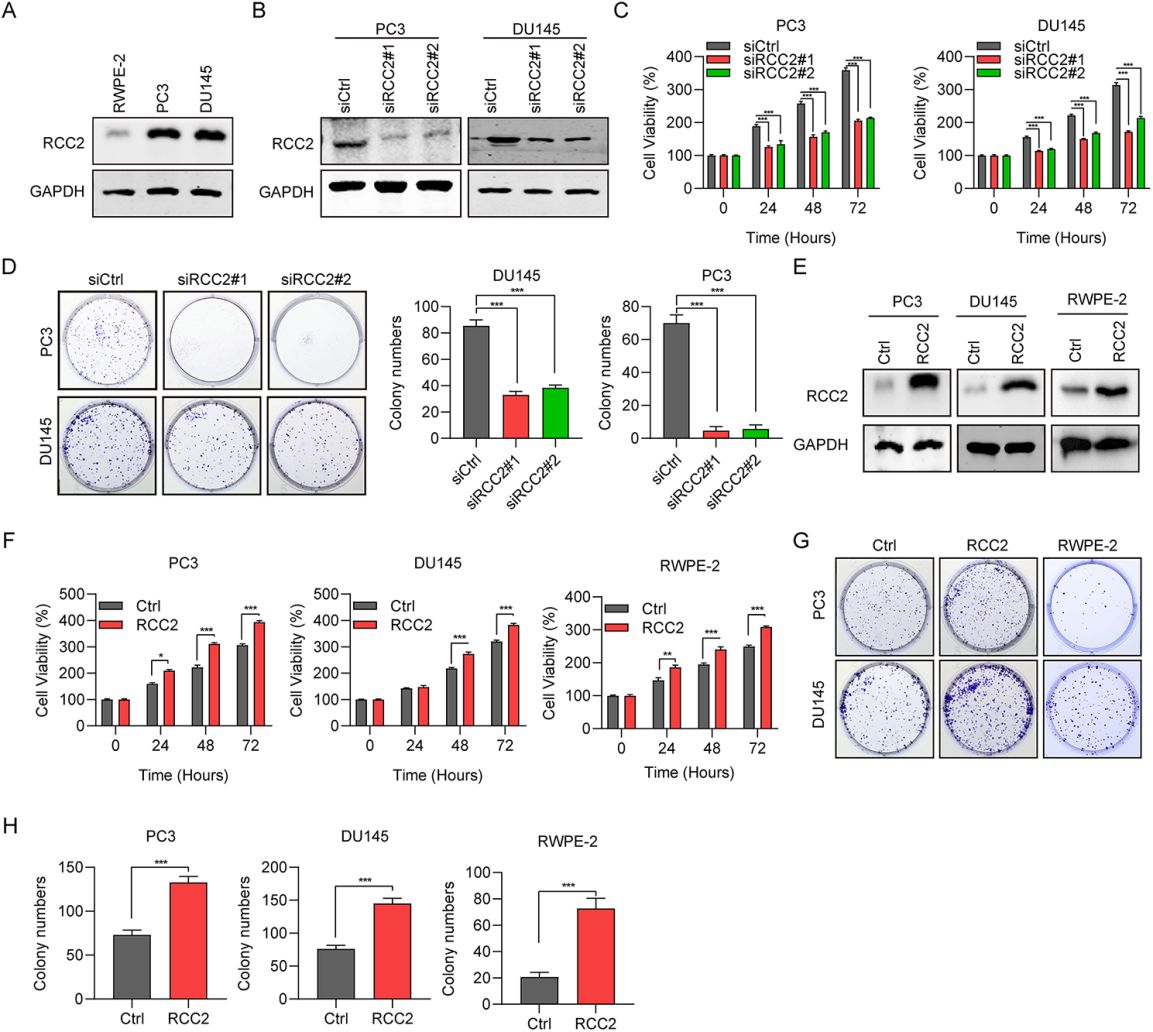
RCC2 promotes cell propagation of prostate cancer. **A** The protein levels of RCC2 in two prostate cancer cell lines (DU145 and PC3) and a normal prostate cell line (RWPE-2) were measured using western blot. **B** The protein levels of RCC2 in DU145 and PC3 cells transfected with siRCC2#1, siRCC2#2, and siCtrl for 48 h were decided with western blot. **C** Cell viability of DU145 and PC3 cells transfected for 24, 48, and 72 h were evaluated using CCK-8 assays. **D** Cell colony generation assays of DU145 and PC3 cells transfected for two weeks. **E** The protein level of RCC2 in DU145, PC3 and RWPE-2 cells transfected with pcDNA-RCC2 and pcDNA-ctrl for 48 h were decided with western blot. **F** Cell viability was assessed using CCK-8 assays. **G** and **H** Cell colony generation ability was detected using cell colony formation assays. *P＜0.05, ***P＜0.001

The expression of RCC2 was up-regulated in PC3 and DU145 cells compared to prostate normal cells RWPE-2 (Fig. 2A). Next, the role of RCC2 on cell propagation in two PCa cells, DU145 and PC3 was investigated. The efficiency of RCC2 knockdown was confirmed using western blot (Fig. 2B). Cell development assays suggested that the knockdown of RCC2 suppressed cell proliferation as shown by CCK-8 (Fig. 2C) and colony development assays (Fig. 2D). Western blot confirmed the overexpression efficiency of RCC2 in PC3, DU145 and RWPE-2 cells (Fig. 2E). In contrast, the ectopic of RCC2 accelerated PCa cell viability (Fig. 2F) and cell colony formation (Fig. 2G H) in PC3, DU145 and RWPE-2 cells. These results showed the mediating role of RCC2 in oncogenic activities in PCa cells in vitro.

### RCC2 promotes cell migration and EMT of prostate cancer

**Fig. 3:**
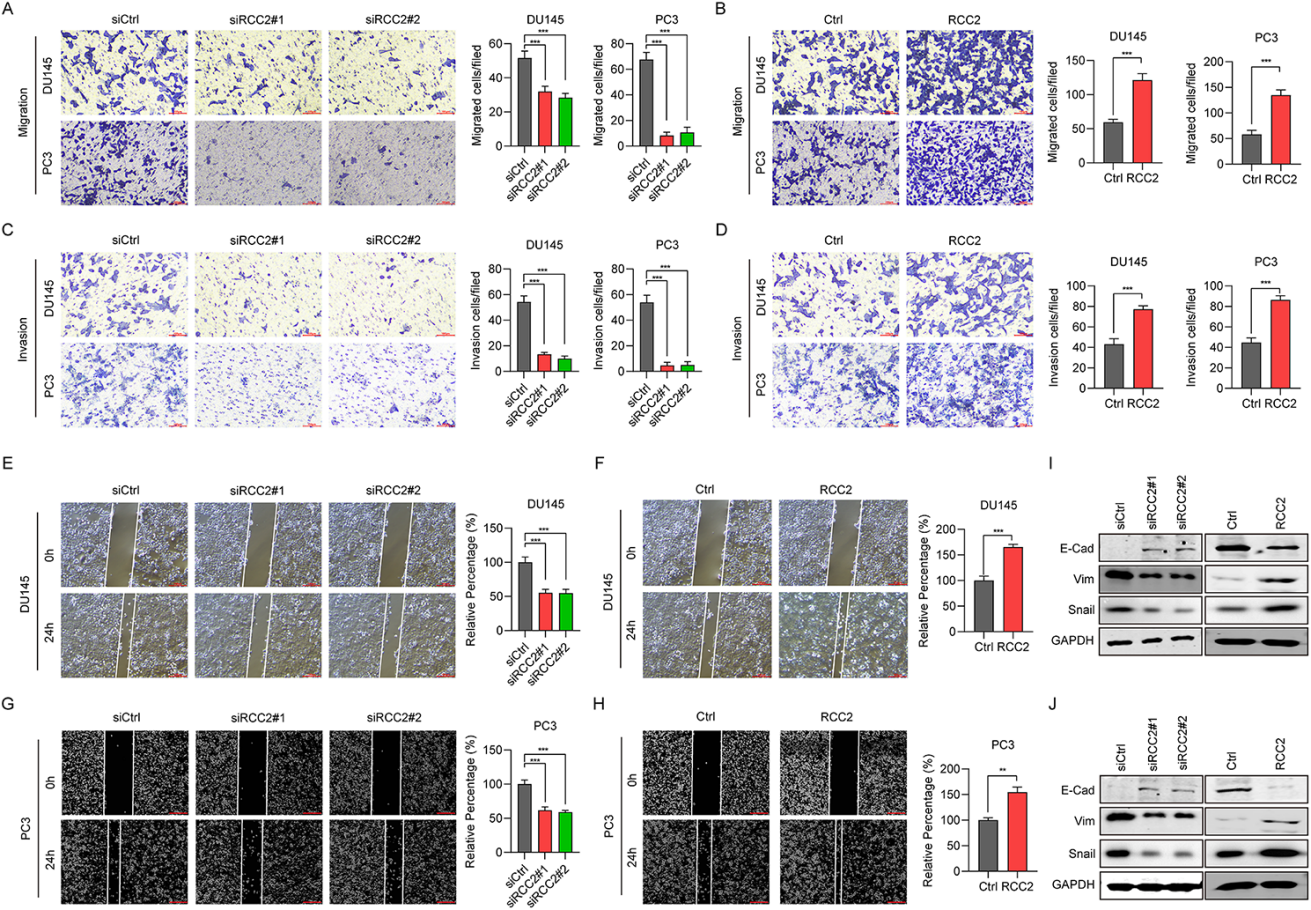
RCC2 drives cell movement and EMT of prostate cancer. **A, B** Representative images and statistics chart of cell movement in DU145 and PC3 cells transfected with pcDNA-RCC2 and pcDNA-ctrl or siRCC2#1, siRCC2#2, and siCtrl for 24 h were evaluated using the Transwell assay. **C, D** Representative images and statistics chart of cell invasion of DU145 and PC3 cells. **E, F, G, H** A wound healing assay was adopted to measure cell migration ability. **I, J** The expression levels of EMT markers, including Snail, E-cad and Vim, in DU145 and PC3 cells. **P＜0.01, ***P＜0.001

Cell movement and invasion assays were used to investigate the role of RCC2 in the cell motility of PCa. According to the movement assay, RCC2 knockdown was associated with less migration of the DU145 and PC3 cells and fewer cells crossing the membrane compared with cells in the siCtrl group (Fig. 3A). By contrast, the overexpression of RCC was associated with increasing cells crossing the membrane in the movement assays in both cell lines (Fig. 3B). A similar trend was indicated by the invasion assay (Fig. 3C, D). Wound-healing assays were used for further verification of the migration ability. The images showed that RCC2 knockdown halted wound closure in both DU145 and PC3 cells at 0-hour or 24-hour incubation (Fig. 3E, G), while RCC2 overexpression facilitated wound closure to some degree (Fig. 3F, H).

EMT is a process where epithelial cells obtain the features of mesenchymal cells, exerting a key effect on PCa [15]. We investigate whether RCC2 regulates EMT through knockdown or overexpression of RCC2. The outcomes indicated that epithelial markers like E-cadherin were up-regulated, whereas mesenchymal markers like Snail and Vim were significantly down-regulated after RCC2 knockdown in DU145 (Fig. 3I) and PC3 cells (Fig. 3J). By contrast, the overexpression of RCC2 promoted EMT progression by increasing Snail, Vim expression and decreasing E-cadherin expression (Fig. 3I, J). Reduced expression of E-cadherin was reported to have a strong association with the progression of PCa [16]. In conclusion, our findings substantiate RCC2 as an oncogene driving the migration, invasion, and EMT of PCa cells.

### Hedgehog signaling pathway participates in RCC2-mediated prostate cancer cells

**Fig. 4:**
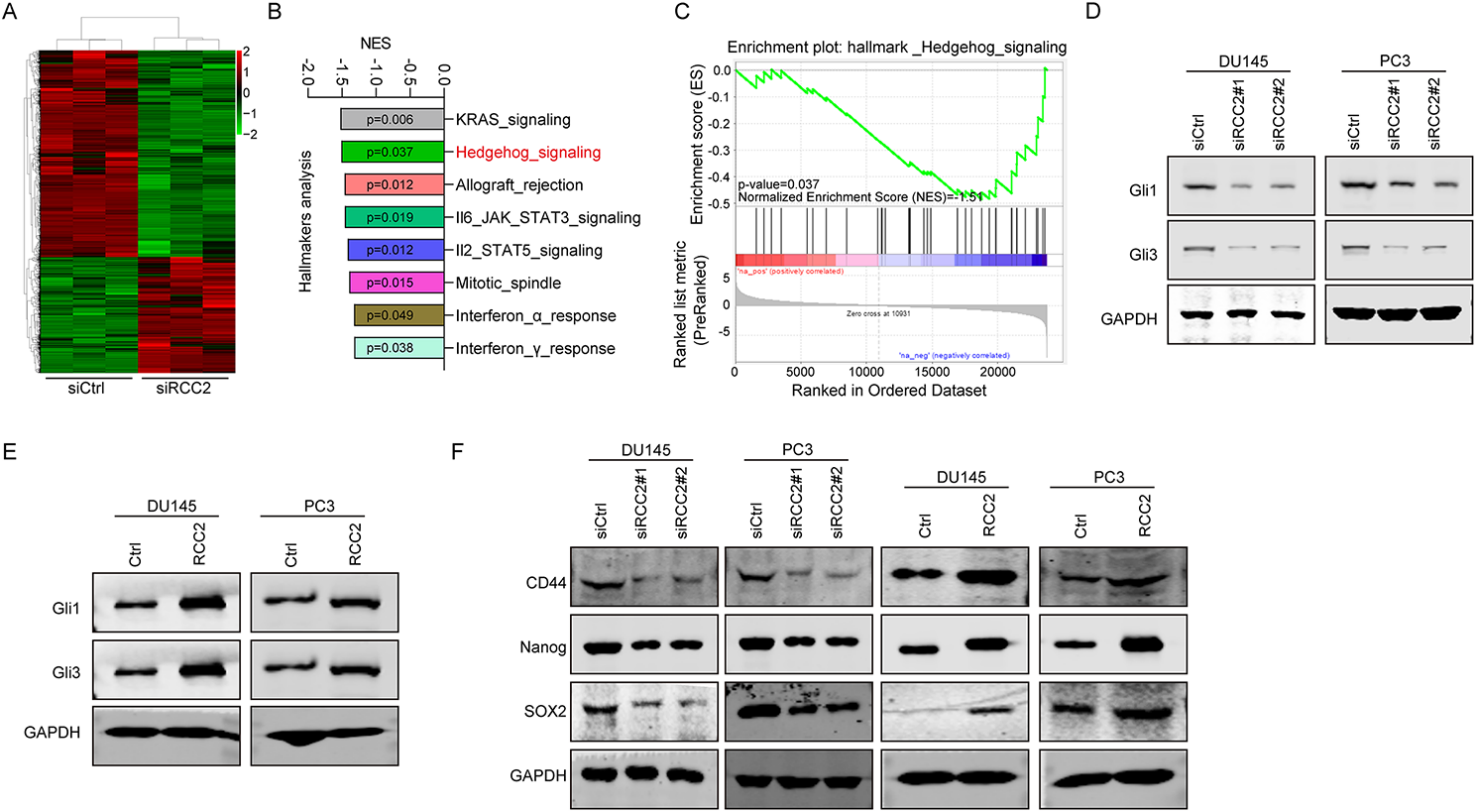
Hedgehog signaling pathway involves in RCC2-mediated prostate cancer cells. **A** Volcanic map showed the DEGs in RCC2 knockdown DU145 cells compared to control DU145 cells. **B** Top 8 signaling were selected in RCC2 knockdown DU145 cells compared to control DU145 cells based on NSE analysis. **C** GSEA analysis showed the Hh signaling pathway was enriched in RCC2 knockdown DU145 cells compared to control DU145 cells. **D, E** The protein expression of three genes (GLI1, GLI3, and SUFU) involved in the Hh signaling pathway in DU145 and PC3 cells after (D) RCC2 knockdown or (E) overexpression were detected with western blot. **F** The protein expression of three CSCs markers (SOX2, Nanog, and CD44) in DU145 and PC3 cells after RCC2 knockdown or overexpression were tested with western blot

To investigate the hidden mechanisms of RCC2 on PCa, overall genome transcriptome analysis was performed on DU145 cells transfected with siRCC2#1. The volcanic map showed there were 938 up-regulated genes and 1669 down-regulated genes in RCC2 knockdown DU145 cells (Fig. 4A). To discover the role of RCC2 on PCa development, we analysis the signaling changes after RCC2 knockdown based on Hall_makers enrichment analysis. Interestingly, we found that KRAS signaling, hedgehog signaling and allograft rejection signaling was most significantly suppressed based on NES (Fig. 4B). We also observed that the Hedgehog (Hh) signaling pathway was the most enriched signaling pathway involved in RCC2 knockdown DU145 cells (Fig. 4C) (NES=-1.51, p < 0.037). There was aberrant Hh pathway signaling in PCa through rendering prostate epithelial cells tumorigenic, promoting the epithelial-to-mesenchymal transition, and making contributions to the growth of castration-resistance, etc., indicating that control of the Hh pathway may be useful in treating recurrent and metastatic PCa [17]. Thus, gene expression levels related to the Hh signaling pathway, such as Gli1 and Gli3 in RCC2 knockdown DU145 and PC3 cells were detected. Western blot results showed that silencing RCC2 led to a decrease in Gli1 and Gli3 levels (Fig. 4D). By contrast, overexpression of RCC2 in DU145 and PC3 cells had the opposite effects (Fig. 4E).

The Hh signaling pathway exerts a crucial effect on the expansion of cancer stem cells (CSCs) [18]. In PCa, NANOG and SOX2 are critical factors that contribute to PCa stem-like cells [19, 20]. Furthermore, the expression of SOX2, Nanog, and CD44 in DU145 and PC3 cells was detected after RCC2 knockdown or overexpression. Compared with siCtrl, silencing RCC2 decreased the protein levels of SOX2, Nanog, and CD44 in DU145 and PC3 cells, while overexpression of RCC2 promoted the protein levels of these three genes (Fig. 4F). These results showed that RCC2 has an oncogenic effect on PCa by increasing the stem ability of tumor cells, which is mediated via Hh signaling pathway.

### RCC2 promotes cell propagation and migration through gli1 in vitro

**Fig. 5:**
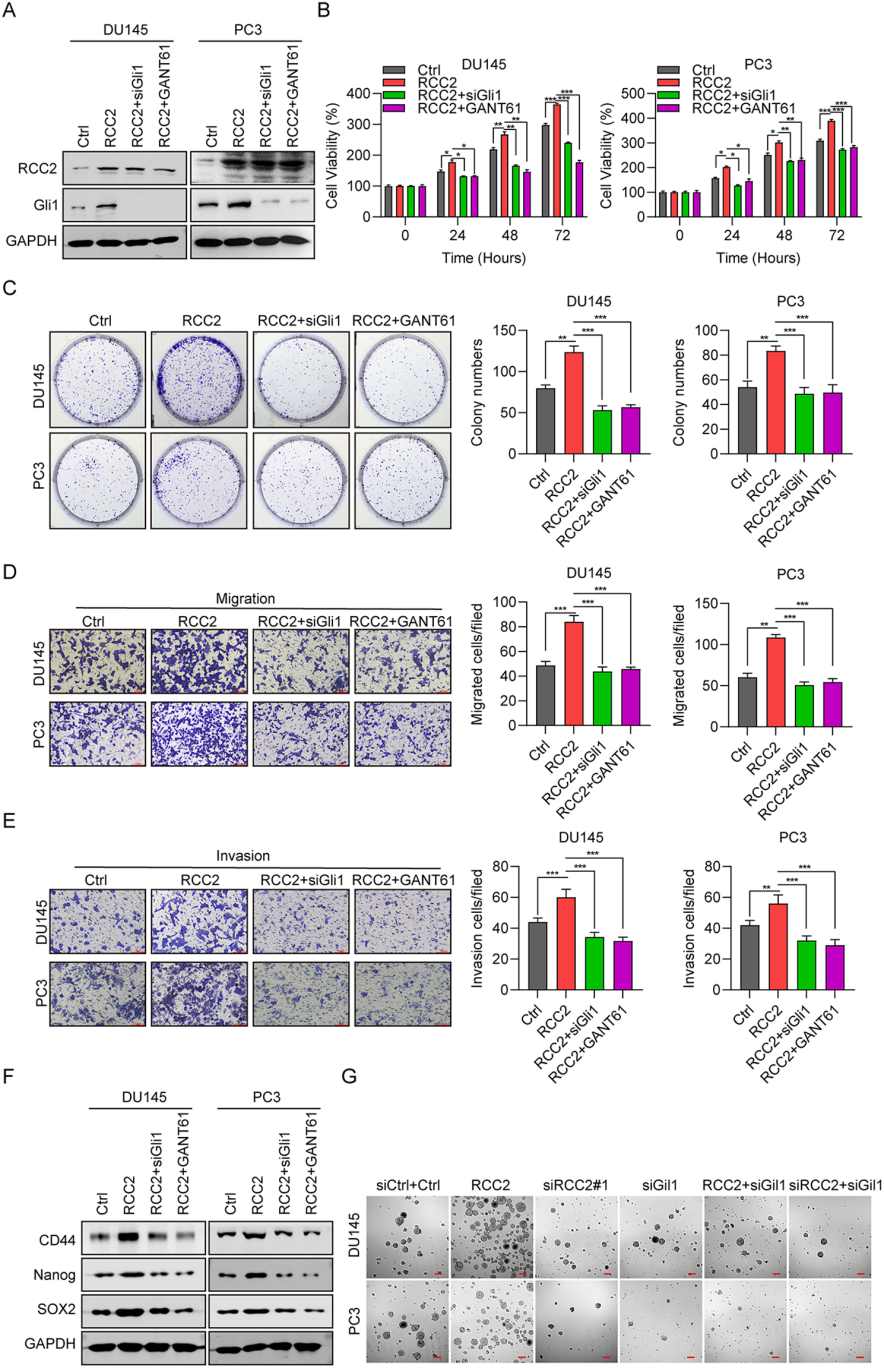
RCC2 promotes cell propagation and migration through GLI1 in vitro. **A** The protein level of RCC2 and Gli1 in DU145 and PC3 cells transfected with Ctrl, RCC2, RCC2 + siGli1, and RCC2 + GANT61, respectively. **B** CCK-8 assay of cell viability in DU145 and PC3 cells between four groups. **C** Cell colony generation assay in DU145 and PC3 cells between four groups. **D** Cell migration ability in DU145 and PC3 cells between four groups. **E** Cell invasion assay in DU145 and PC3 cells between four groups. **F** The expression of CSC marker genes, including SOX2, Nanog, CD44. *P < 0.05, **P < 0.01, ***P < 0.001. **G**. The effect of RCC2 overexpression or knock down on the capacity of oncosphere formation. Representative images. Scale bar, 100 μm

siGli1 and a Gli inhibitor (GANT61) were used to confirm the effect of Gli1 on DU145 and PC3 cells. The inhibitory effect of siGli1 and GANT61 were confirmed using western blot, with significantly decreased Gli1 levels in DU145 and PC3 cells (Fig. 5A). Subsequently, the role of Gli1 in cell propagation was investigated with CCK8 assay and cell colony formation assay. The results showed that siGli1 and GANT61 significantly inhibited cell development of DU145 and PC3 cells (Fig. 5B, C). The role of Gli1 in cell motility was examined using migration and invasion assays. It was observed that siGli1 and GANT61 significantly decreased the number of cells crossing the membrane in both the movement and invasion assays in DU145 and PC3 cells (Fig. 5D, E). According to western blot outcomes, the protein level of SOX2, Nanog, and CD44 in DU145 and PC3 cells were significantly decreased in RCC2 + siGli1 and RCC2 + GANT61 groups compared to the RCC2 group, indicating that silencing Gli1 reversed the role of RCC2 in CSCs (Fig. 5F). In addition, we observed that RCC2 overexpression dramatically increased the capacity of oncosphere formation, while RCC2 or Gli1 knock down significantly decreased the capacity of oncosphere formation (Fig. 5G). These results indicated that RCC2 exerted its oncogenic effect on PCa by increasing the stem ability of tumor cells, which is regulated via Gli1 and activation of the Hh signaling pathway.

### RCC2 promotes tumor growth by promoting gli1 in vivo

**Fig. 6:**
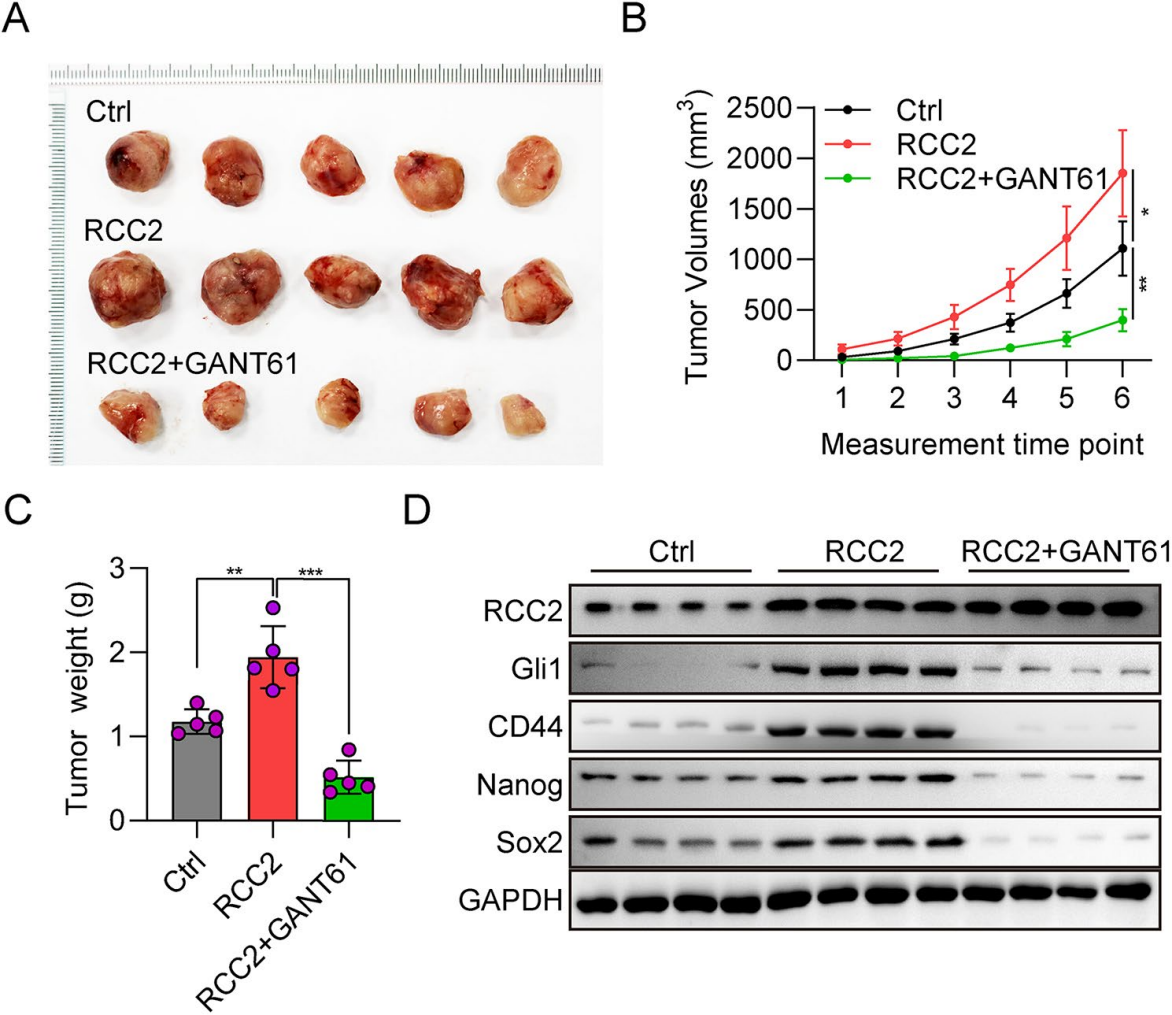
RCC2 drives tumor development by promoting Gli1 in vivo. **A** Figs of mouse xenograft tumors from the Ctrl, RCC2 overexpression, and RCC2 overexpression + GANT61 groups. **B** Tumor growth curves in mice among three groups. **C** Tumor weight of mice among three groups. **D** The protein expression of RCC2, Gli1, SOX2, Nanog, and CD44 in mice among three groups were tested with western blot. *P＜0.05, **P＜0.01, ***P＜0.001

To evaluate the role of RCC2 and Gli1 in vivo, a xenograft tumor mouse was prepared by injecting DU145-RCC2 or DU145-RCC + GANT61 cells into the mice axillae. As indicated in Fig. 6A–C, the tumor size, weight, and volumes were significantly increased in DU145-RCC2 xenograft tumors compared to the control groups, which showed the oncogenic role of RCC in tumor growth. Compared with the DU145-RCC2 group, the tumor size and tumor volume in the DU145-RCC2 + GANT61 group were significantly decreased, indicating that inhibiting Gli1 reversed the effect of RCC on tumor growth. In other words, RCC2 promotes tumor growth by promoting Gli1. We also tested the expression of SOX2, Nanog, and CD44 in three tumor model groups. The findings demonstrated that the protein level of SOX2, Nanog, and CD44 were increased in RCC2 overexpressed mice but decreased in RCC2 + GANT61 treated mice (Fig. 6D). These in vivo findings were consistent with the in vitro findings, confirming that RCC2 exerted its oncogenic role through Gli1 and activation of the Hh signaling pathway.

## Discussion

RCC2 has been reported to participate in the control of cell cleavage, which is required for the proper completion of mitosis [21]. There are growing reports on the role of RCC2 in human tumor growth, apoptosis, EMT, and drug resistance. For example, RCC2 exerts an oncogenic effect on breast cancer by activating the Wnt-signaling pathway, thus driving cell propagation and movement through EMT [8]. The overexpression of RCC2 occurred in lung cancer and ovarian cancer, and their sensitivity to drug-induced cell death was altered by RCC2 expression in cancer cells, indicating that RCC2 may function as an effective marker for forecasting chemotherapeutic reaction [10]. RCC2 promotes radio-resistance and propagation in glioblastoma by transcriptionally stimulating DNMT1 in a STAT3-dependent manner [22]. In hepatocellular carcinoma (HCC), increased expression of RCC2 facilitated HCC cell invasion and chemoresistance to cisplatin [12]. These reports indicate that RCC2 is an oncogene in various tumors that drives cancer cell progression, metastatic actions, and therapeutic resistance in tumor cells.

This research found the up-regulation of RCC2 in PCa tissues from the TCGA database, consistent with the report of Li et al. [23]. Additionally, the promoter methylation level of RCC2 was down-regulated in PCa samples. High expression of RCC2 indicated advanced regional lymph node metastasis and shorter DFS. Furthermore, we used “gain of function” and “loss of function” assays in vitro to study the effect of RCC2 on PCa, and the results of the movement and invasion assays demonstrated that RCC2 facilitated cell propagation, movement, invasion, and EMT in two PCa cell lines DU145 and PC3, indicating the oncogenic role of RCC2 in PCa. In order to explore the regulatory mechanism, we performed whole genome transcriptome analysis using RNA-seq in RCC2 knockdown DU145 cells. A total of 2607 DEGs were found, and the GSEA results showed that the Hh signaling pathway was ranked first.

The Hh signaling pathway was initially observed in Drosophila as a main regulating agent of segment patterning in growth [24], which exerts a key effect on a lot of basic processes, such as embryonic growth, tissue homeostasis, neoplastic transformations, and malignant tumors by controlling cancer cell propagation, malignancy and metastasis, and the growth of cancer stem cells [18]. Gli1 acts as a core mediating agent of the Hedgehog pathway that strongly associated with prognosis in prostate cancer [25]. Gli3, a member of the GLI family of transcription elements, is also a core sonic hedgehog (SHH) signaling pathway effector, required and adequate for the development and movement of AR-positive PCa cells [26]. Therefore, to confirm the role of the Hh signaling pathway on PCa, we detected the protein levels of these three genes in RCC2 knockdown or overexpressed DU145 cells. The findings revealed that silencing RCC2 led to reduced Gli1 and Gli3 levels, while overexpression of RCC2 had the opposite results.

Gli1 is also a potential cancer stem cells (CSC) marker in PCa [25]. CSCs are related to a given set of surface antigens, including CD44+, to initiate tumors [27]. CD44, a multifunctional protein, participates in cell adhesion and signaling [28]. The enrichment of greatly purified CD44 + PCa cells from xenograft human tumors is made in tumorigenic and metastatic progenitor cells [28]. In PCa, NANOG-a key pluripotency reprogramming element, contributes to PCa stem-like cells [19]. It was reported that the SRY-related HMG-box gene 2 (SOX2) is responsible for the epigenetic reprogramming of prostate stem cells (PSCs) toward prostate cancer stem cells (PCSCs) in PCa [20]. SOX2 promotes tumorigenesis and the anti-apoptotic nature of human PCa cells [29]. Furthermore, the expression of SOX2, Nanog, and CD44 in DU145 and PC3 cells was detected after RCC2 knockdown or overexpression, and the results showed that silencing RCC2 decreased the protein levels of SOX2, Nanog, and CD44 in DU145 and PC3 cells, while the protein levels were increased by overexpression of RCC2. These results also confirmed that RCC2 exerted stem cell ability through the Hh/GLI1 signaling pathway.

To further confirm our results, recovery assays were conducted using siGli1 and GANT61 (a GLI inhibitor) in RCC2 overexpressed DU145 and PC3 cells. The results of the CCK8 assay and cell colony formation assay showed that suppressing Gli1 or GL1 family members in DU145 and PC3 cells recovered the cell propagation to normal levels, which were previously significantly increased by RCC2 overexpression. Cell migration and invasion assays results also got similar findings. The expression levels of three stem markers were detected using western blot, which showed that inhibiting Gli1 decreased the protein levels of SOX2, Nanog, and CD44 in DU145 and PC3 cells compared to the RCC2 group. Finally, animal experiments were performed in vivo to verify our findings. We found that tumor size and tumor weight were significantly increased in RCC2 overexpressed mice but decreased to normal levels after inhibiting with Gli1. Western blot of SOX2, Nanog, and CD44 expression showed the similar tread, which increased after RCC2 overexpression, and decreased after RCC2 knockdown.

In conclusion, we found that RCC2 has an oncogenic role in PCa, which is adjusted via triggering Gli1 and stimulating Hh signaling pathway. Our research emphasizes a new effect and a novel administrative mechanism of RCC2 in PCa progression.
